# Association between life’s essential 8 and cognitive impairment in older patients: results from NHANES 2011–2014

**DOI:** 10.1186/s12877-024-05547-4

**Published:** 2024-11-14

**Authors:** Hui Wang, Sensen Wu, Dikang Pan, Yachan Ning, Cong Wang, Jianming Guo, Yongquan Gu

**Affiliations:** Department of Vascular Surgery, Xuanwu Hospital, Capital Medical University, No. 45, Changchun Street, Beijing, Beijing, 100053 China

**Keywords:** Life’s essential 8, NHANES, Cardiovascular health, Cognitive impairment

## Abstract

**Background:**

This study aimed to examine the association between the American Heart Association’s (AHA) newly revised Life’s Essential 8 (LE8) algorithm, designed for assessing cardiovascular health (CVH), and cognitive impairment among older adults in the United States.

**Methods:**

This study employed a cross-sectional design, utilizing data from the 2011–2014 National Health and Nutrition Examination Survey to explore the relationship between CVH and cognitive impairment in older adults. CVH scores are assessed based on the AHA definition of the LE8, categorized into three tiers: low (0–49), medium (50–79), and high (80–100). Cognitive impairment is evaluated using three distinct scoring systems: the Consortium to Establish a Registry for Alzheimer’s Disease (CERAD), the Animal Fluency Test (AFT), and the Digit Symbol Substitution Test (DSST). The lowest quartile as the cut-off point; below or equal to the lower quartile was considered as low cognitive population, and above the lower quartile was normal population. To analyze the association, multivariable logistic regression and restricted cubic spline (RCS) models were employed.

**Results:**

A significant negative correlation exists between the LE8 and cognitive impairment. After adjusting for multiple variables, the odds ratios (OR) for cognitive impairment, as measured by the CERAD, AFT, and DSST, were compared between patients with high and low CVH. The results indicated OR values of 0.60 (95% CI: 0.36–0.98), 0.72 (95% CI: 0.52–0.97), and 0.29 (95% CI: 0.16–0.53) for the CERAD, AFT, and DSST, respectively. Additionally, the RCS curve demonstrated a significant linear relationship between lifestyle factors encapsulated by the LE8 and cognitive impairment.

**Conclusions:**

The findings indicate higher adherence to LE8 was associated with lower odds of cognitive impairment. Furthermore, maintaining optimal CVH is crucial in preventing cognitive impairment.

**Supplementary Information:**

The online version contains supplementary material available at 10.1186/s12877-024-05547-4.

## Introduction

As the global population ages, cognitive impairment emerges as a critical health concern among the elderly. Projections suggest that by 2060, the United States will witness over 21.55 million cases of dementia. A considerable portion of these patients may experience a decline leading to death due to cognitive impairment [1, 2]. Notably, research indicates that elderly individuals with cognitive impairment, resulting from non-Alzheimer’s causes, suffer from markedly reduced quality of life [3]. Extensive studies have established a strong correlation between cognitive function and cardiovascular health (CVH), identifying cardiovascular diseases as the second leading cause of cognitive impairment, accounting for 15–30% of cases [4–7]. Approximately one-third of cardiovascular disease patients demonstrate various levels of cognitive impairment. Therefore, a deeper understanding of the interplay between CVH and cognitive impairment is essential for enhancing the prevention and management of cognitive impairment in these individuals [8].

In 2010, the AHA formulated a strategy aimed at enhancing population and individual health, introducing “Life’s Simple 7” as a metric for cardiovascular health assessment. Subsequent research, however, revealed certain inadequacies in this model, including a limited scope of health behaviors and a rudimentary scoring mechanism [9].To address these limitations, the AHA revised the indicator in 2022, introducing LE8. This updated version, in comparison to its predecessor Life’s Simple 7, incorporates a new sleep indicator, thereby offering a more inclusive and nuanced scoring system that better accommodates individual variations. LE8 delineates CVH with greater breadth and precision than its antecedent [10, 11].Extensive research has demonstrated a significant correlation between the LE8 score and several health outcomes, including cardiovascular diseases, all-cause mortality, and a range of complications associated with vascular diseases [12–14]. Presently, there is a lack of research employing LE8 as a biomarker to explore the association between CVH and cognitive impairment.

We hypothesize a significant association between the LE8 score and cognitive impairment in elderly patients. Consequently, this cross-sectional study employs data from the National Health and Nutrition Examination Survey (NHANES) database to explore the relationship between the LE8 score, a marker of CVH, and cognitive impairment in American patients aged 60 years and older.

## Methods

### Study data and study population

The data for this study were sourced from the NHANES database. NHANES, established in 1999, is a research initiative that evaluates the health and nutritional status of adults and children across the United States. This program systematically selects a representative sample of approximately 10,000 individuals biennially. The survey encompasses detailed data on demographics, socioeconomic factors, dietary habits, medical history, laboratory examinations, and assorted health-related concerns. It employs a sophisticated, stratified, multi-stage sampling methodology, augmented by sample weights, to precisely estimate the prevalence of diverse diseases [15, 16].

**Fig. 1:**
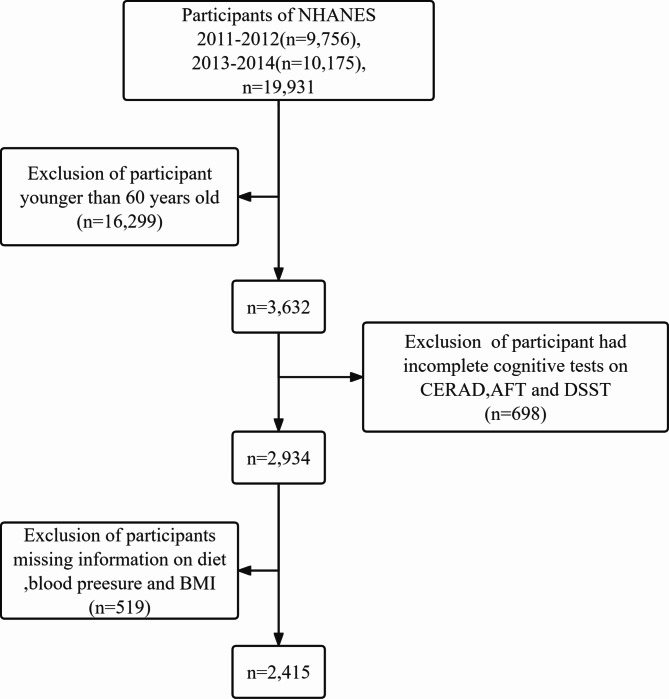
Selection of study population

This analysis encompasses data from participants in the 2011–2012 and 2013–2014 biennial cycles of the NHANES. These specific cycles were selected due to the inclusion of three cognitive assessments: the CERAD, AFT, and DSST. Our study was confined to patients aged 60 and above who underwent cognitive function assessments, encompassing 2934 individuals initially. Exclusions were made for patients with incomplete datasets, specifically missing information on diet, blood pressure, and BMI. Consequently, the final analysis included 2654 patients, as detailed in Fig. 1.

This study adhered to the Strengthening the Reporting of Observational Studies in Epidemiology (STROBE) reporting guidelines [17] and involved a secondary analysis of de-identified, publicly available data from NHANES. Consequently, it was exempt from additional institutional review board approval and informed consent requirements.

### Measurement of LE8 score

The LE8 score comprises eight metrics, encompassing four behavioral (diet, physical activity, tobacco/nicotine exposure, and sleep health) and four biological factors (body mass index [BMI], non-HDL cholesterol, blood glucose, and blood pressure). The NHANES database provides the specific algorithm for computing the LE8 score for each metric, as detailed in Supplementary Table 1. The CVH metrics are scored on a scale ranging from 0 to 100 points. The aggregate LE8 score is derived by calculating the arithmetic mean of these eight metrics. Within this study, an LE8 score between 80 and 100 denotes high CVH, 50 to 79 signifies moderate CVH, and 0 to 49 indicates low CVH, with lower scores reflecting poorer health status and higher scores indicating superior health [10, 11].

Dietary indicators were evaluated using the Healthy Eating Index (HEI) 2015. The HEI-2015’s components and scoring criteria are detailed in Supplementary Table 2. Dietary intake data, gathered through two 24-hour dietary recalls, were merged with the United States Department of Agriculture (USDA) food pattern equivalent database to compute the HEI-2015 scores [18]. Data pertaining to physical activity, nicotine exposure, and sleep status are collectable via questionnaires. Laboratory data provide information on blood indices, glycated hemoglobin, and fasting blood glucose levels. Additionally, blood pressure and BMI measurements are available in examination data.

### Cognitive performance assessment

Cognitive performance was assessed through a structured questionnaire, which included the following components: (1)CERAD; (2)AFT; (3)DSST [19]. The CERAD Word Learning Test is specifically tailored to measure verbal learning and memory capacities. This test consists of three consecutive trials in which participants are asked to read aloud and subsequently recall a list of 10 unrelated words. This is followed by a delayed recall test conducted approximately 10 min later. The maximum achievable score for each trial is 10 points, leading to an aggregate maximum score of 40 points when including the three initial trials and the delayed recall. AFT evaluates verbal category fluency, which is a component of executive function, and also assesses semantic memory and processing speed. During this test, participants are tasked with enumerating as many animals as possible within a one-minute timeframe, with each unique animal mentioned being awarded one point. The DSST serves as a multifaceted metric for cerebral health, evaluating processing speed, visual scanning, sustained attention, and working memory. During the assessment, participants are required to utilize a reference key to correctly associate symbols with corresponding numbers, completing 133 such pairings within a two-minute period. However, there are no recognized thresholds for the DSST, CERAD, and AFT to distinguish cognitive impairment. Age is acknowledged as a substantial confounding variable in cognitive test outcomes. Consequently, participants were categorized by age groups: 60 to less than 70 years, 70 to less than 80 years, and 80 years and older. Cognitive impairment was identified using the 25th percentile of the age-stratified scores as the cutoff, with values below the 25th percentile (P25) being indicative of cognitive dysfunction [20].

### Covariates assessment

In the current study, the covariates analyzed included a range of demographic factors: age; gender, with categories for male and female; race and ethnicity, which encompassed non-Hispanic black, non-Hispanic white, Mexican-American, other Hispanic, and additional races, such as multiracial; marital status, segmented into married/cohabiting, never married, and widowed/divorced/separated; poverty income ratio (PIR), stratified into low income (below 1.30), middle income (1.30–3.49), and high income (3.50 and above); educational attainment, categorized as less than high school, high school graduate, and college or higher; smoking status, where individuals who have smoked 100 or more cigarettes in their lifetime were classified as smokers; alcohol consumption, with those drinking 12 or more times per year designated as heavy drinkers; and self-reported medical conditions including myocardial infarction, angina, heart failure, and cardiovascular disease.

### Statistical analysis

Considering the intricate sampling framework of the NHANES, this study incorporated sample weights, clustering, and stratification within all analyses to yield estimates representative of the national population. Categorical variables are depicted as counts and respective weighted proportions, with their associations evaluated via the Rao-Scott chi-square test. The distribution of continuous variables, whether normal or non-normal, is determined using the Kolmogorov-Smirnov normality test. Variables that do not follow a normal distribution are reported as weighted medians and interquartile ranges (IQRs), whereas normally distributed continuous variables are described using weighted means and standard errors (SEs), with the latter estimated using the Taylor series linearization method.

Multiple linear regression models were used to investigate the association between LE8 and three cognitive function tests. Model 1 was unadjusted; Model 2 was a crude model adjusted for age and gender. Model 3 further adjusted for race, marital status, education level, poverty income ratio (PIR), relevant laboratory indicators, and comorbid diseases. In this study, LE8 was analyzed as both a continuous and categorical variable. When LE8 was treated as a continuous variable, RCS with four knots at the 5th, 25th, 75th, and 95th percentiles were employed to explore the potential dose-response relationship between LE8 and the three different cognitive function tests, as well as overall cognitive function, adjusting for all covariates, with the median serving as the reference point for all participants. To assess whether subpopulations in artificial statistics might affect the results, subgroup analyses were conducted by gender, age, race, marital status, PIR, diabetes, hypertension, and cardiovascular diseases. The P-values for the interaction terms between the LE8 score and stratifying factors were used to evaluate the significance of the interactions.

All statistical analyses were conducted using R (version 4.3.1) and SPSS (version 26.0, IBM Corp., USA). A two-tailed P-value of less than 0.05 was considered to indicate statistical significance.

## Results

### Baseline characteristics of participants

In this study, 19,931 participants were included in the NHANES from 2011 to 2014. After excluding patients younger than 60 years old, those who did not undergo cognitive function testing, and patients who had undergone cognitive testing but had missing key data, a total of 2,415 patients were included in the final analysis. Table 1 delineates the baseline demographic and clinical characteristics of the study cohort. The mean age was 69.44 ± 6.77 years, with males constituting 50.23% of the sample. The average LE8 score, along with their standard deviations, for the low, middle, and high CVH groups were 44.53 ± 4.54, 65.43 ± 8.13, and 84.39 ± 3.57, respectively. Cognitive impairment, as assessed by the CEARD, AFT, and DSST metrics, was identified in 529 (21.9%), 467 (19.34%), and 549 (22.73%) participants, respectively. Notable statistical discrepancies were observed across the LE8 subgroups concerning variables such as BMI, race, marital status, education, income, smoking habits, insomnia, depression, cardiovascular diseases, hypertension, hyperlipidemia, white blood cell count, red blood cell count, and others, all reaching statistical significance (*P*<0.05).

**Table 1:** Baseline characteristic of the study population by cardiovascular health (CVH) status

Characteristic	Total (*n* = 2415)	Low CVH (0–49) (*n* = 277)	Moderate CVH (50–79) (*n* = 1867)	High CVH (80–100) (*n* = 271)	*P* value
Age, y	69.44 ± 6.77	68.55 ± 6.52	69.53 ± 6.79	69.74 ± 6.79	0.057
Sex, n (%)					0.504
Male	1213 (50.23)	130 (46.93)	945 (50.62)	138 (50.92)	
Female	1202 (49.77)	147 (53.07)	922 (49.38)	133 (49.08)	
BMI	29.06 ± 6.32	33.73 ± 7.53	28.95 ± 5.95	24.64 ± 3.36	< 0.001
Race, n (%)					< 0.001
Mexican American	199 (8.24)	28 (10.11)	155 (8.30)	16 (5.90)	
Other Hispanic	252 (10.43)	29 (10.47)	207 (11.09)	16 (5.90)	
Non-Hispanic White	1207 (49.98)	116 (41.88)	931 (49.87)	160 (59.04)	
Non-Hispanic Black	549 (22.73)	96 (34.66)	415 (22.23)	38 (14.02)	
Other Race - Including Multi- Racial	208 (8.61)	8 (2.89)	159 (8.52)	41 (15.13)	
Marital status, n (%)					< 0.001
Married/Living with Partner	1424 (58.96)	138 (49.82)	1099 (58.86)	187 (69.00)	
Never married	142 (5.88)	21 (7.58)	107 (5.73)	14 (5.17)	
Widowed/Divorced/ Separated	849 (35.16)	118 (42.60)	661 (35.40)	70 (25.83)	
Education levels, n (%)					< 0.001
< High school	596 (24.68)	88 (31.77)	477 (25.55)	31 (11.44)	
High school	566 (23.44)	79 (28.52)	448 (24.00)	39 (14.39)	
College or above	1253 (51.88)	110 (39.71)	942 (50.46)	201 (74.17)	
Income status					< 0.001
High income	691 (28.61)	115 (41.52)	532 (28.49)	44 (16.24)	
Middle income	923 (38.22)	102 (36.82)	741 (39.69)	80 (29.52)	
Low income	801 (33.17)	60 (21.66)	594 (31.82)	147 (54.24)	
Smoking, n (%)	1229 (50.89)	197 (71.12)	948 (50.78)	84 (31.00)	< 0.001
Drinking, n (%)	1669 (69.11)	192 (69.31)	1284 (68.77)	193 (71.22)	0.716
Sleep Disorder, n (%)	715 (29.61)	140 (50.54)	541 (28.98)	34 (12.55)	< 0.001
Depression, n (%)	231 (9.57)	42 (15.16)	179 (9.59)	10 (3.69)	< 0.001
Stroke, n (%)	160 (6.63)	23 (8.30)	126 (6.75)	11 (4.06)	0.123
Cardiovascular diseases, n (%)	433 (17.93)	65 (23.47)	333 (17.84)	35 (12.92)	0.005
Hypertension, n (%)	1480 (61.28)	209 (75.45)	1171 (62.72)	100 (36.90)	< 0.001
Triglycerides (mg/dL)	124.74 ± 54.00	156.55 ± 66.64	124.83 ± 51.38	91.59 ± 33.89	< 0.001
Cholesterol (mg/dL)	191.53 ± 43.00	203.92 ± 53.96	190.28 ± 42.00	187.53 ± 34.32	< 0.001
WBC	6.98 ± 2.52	8.00 ± 3.51	6.95 ± 2.40	6.15 ± 1.62	< 0.001
RBC	4.50 ± 0.50	4.60 ± 0.56	4.49 ± 0.49	4.44 ± 0.47	0.002
LE 8	65.16 ± 12.05	44.53 ± 4.54	65.43 ± 8.13	84.39 ± 3.57	< 0.001
CERAD, n (%)	529 (21.9)	62 (22.38)	432 (23.14)	35 (12.92)	< 0.001
AST, n (%)	467 (19.34)	65 (23.47)	356 (19.07)	46 (16.97)	0.130
DDST, n (%)	549 (22.73)	89 (32.13)	441 (23.62)	19 (7.01)	< 0.001
Global cognitive impairment, n (%)	998 (41.33)	138 (49.82)	782 (41.89)	78 (28.78)	< 0.001

WBC: White blood cells; RBC: Red blood cells; The Consortium to Establish a Registry for Alzheimer’s Disease (CERAD); Digit Symbol Substitution Test (DSST); Animal Fluency test (AFT);

Continuous variables were shown in mean (SD) and categorical variables were shown in percentages

### The association between LE8 and cognitive impairment

Logistic regression analyses were utilized to examine the relationship between the LE8 score and cognitive function among participants, as presented in Table 2. The findings revealed that a higher CVH score consistently correlated with a lower risk of cognitive impairment in the unadjusted model 1, the partially adjusted model 2 (which controlled for gender and age), and the fully adjusted model 3, which considered additional confounding factors, including age, gender, race, marital status, educational attainment, income level, smoking habits, sleep disorders, depression, cardiovascular diseases, hypertension, triglyceride levels, cholesterol levels, and blood cell counts. Relative to low CVH, moderate CVH demonstrated statistically significant improvements in the DSST scores across all three analytical models. However, this association was not observed in the CERAD scores, and the AFT scores were only significant in the first two models. Additionally, when analyzed as a continuous variable in logistic regression models, the LE8 score inversely correlated with the risk of cognitive impairment, indicating a decrease in risk with higher LE8 score.

**Table 2:** Association of the life’s essential 8 scores with cognitive impairment of three different scores s in the multivariate linear regression model

Variables	CERAD test score			Animal Fluency Test score			DSST score		
	Model 1	Model 2	Model 3	Model 1	Model 2	Model 3	Model 1	Model 2	Model 3
LE8 score	**0.87** (0.81–0.95) **	**0.86** (0.79–0.93) **	**0.88** **(0.80–0.97)** *	**0.87** (0.80–0.94) **	**0.86** (0.79–0.94) **	1.01 (0.91–1.12)	**0.73** **(0.68–0.79)** **	**0.73** **(0.67–0.79)** **	**0.87** **(0.78–0.98)** *
LE8 score, (group)									
Low CVH	1.00 (Reference)	1.00 (Reference)	1.00 (Reference)	1.00 (Reference)	1.00 (Reference)	1.00 (Reference)	1.00 (Reference)	1.00 (Reference)	1.00 (Reference)
Moderate CVH	0.97 (0.77–1.32)	0.96 (0.73–1.26)	0.94 (0.68–1.30)	**0.76** (0.58–0.99) *	**0.74** (0.57–0.97) *	0.87 (0.63–1.21)	**0.62** (0.49–0.80) **	**0.61** (0.48–0.78) **	**0.71** (0.50–0.99) *
High CVH	**0.62** (0.41–0.92) *	**0.57** (0.38–0.85) **	**0.60** (0.36–0.98) *	**0.65** (0.44–0.95) *	**0.63** (0.43–0.92) *	**0.72** (0.52–0.97) *	**0.18** (0.12–0.29) **	**0.18** (0.11–0.28) **	**0.29** (0.16–0.53) **

Data are presented as β (95% confidence intervals). * *P* < 0.05. ** *P* < 0.01

LE8, Life’s Essential 8; OR, odds ratio; CI, confidence interval; Registry for Alzheimer’s Disease (CERAD); Digit Symbol Substitution Test (DSST); Animal Fluency test (AFT)

Model 1: unadjusted model

Model 2: adjusted for Age, Sex

Model 3: adjusted for Age, Sex, Race, Marital status, Education levels, Income status, Smoking, Sleep Disorder, Depression, Cardiovascular diseases, Hypertension, Triglycerides, Cholesterol, White blood cells, red blood cells

### Subgroup analyses of factors impacting the association of LE8 with cognitive impairment

Supplementary Tables 3–5 present a stratified subgroup analysis examining the relationship between LE8 score and cognitive dysfunction, utilizing three distinct cognitive function assessments and accounting for variables including age, gender, race, income, marital status, hypertension, cardiovascular disease, and insomnia. The influence of LE8 score on cognitive dysfunction was stable across all predetermined subgroups, with all interaction *P*>0.05.

### Dose-response analysis of LE8 score with cognitive impairment

**Fig. 2:**
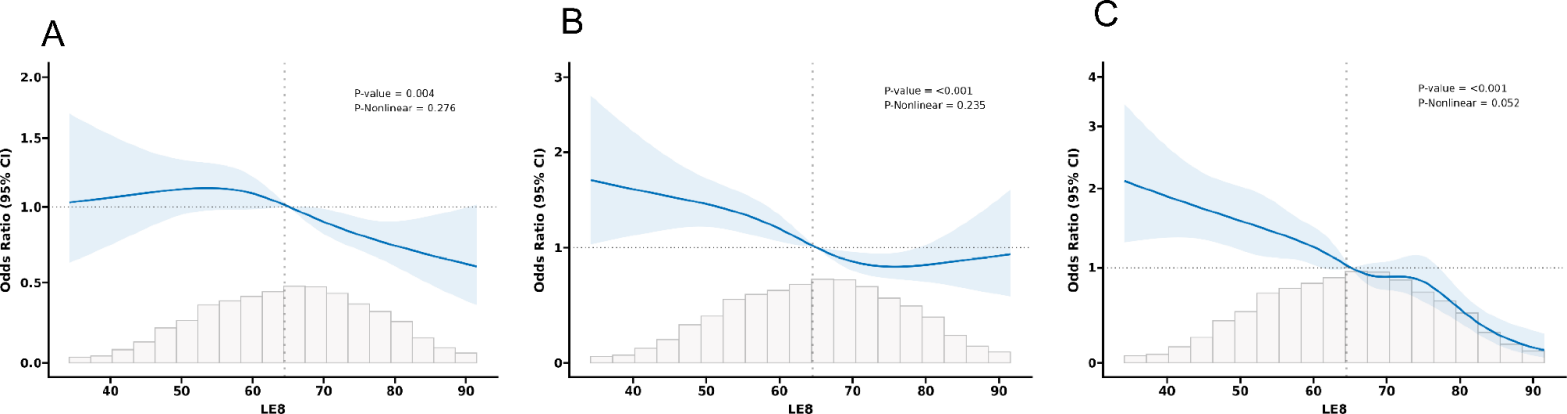
Association of Life’s Essential 8 score with cognitive impairment in a restricted cubic spline model A: The Consortium to Establish a Registry for Alzheimer’s Disease (CERAD), B: The Animal Fluency Test (AFT) C: The Digit Symbol Substitution Test (DSST)

Analysis using RCS indicates a general association between the LE8 score and cognitive impairment, yet the relationship is not non-linear, as evidenced by the DSST score (P for non-linearity = 0.276), AFT (P for non-linearity = 0.235), and CERAD test results (P for non-linearity = 0.052) (Fig. 2). The LE8 score demonstrates a favorable correlation with cognitive impairment at a minimum threshold score of 65(with an estimated OR of 1). Using 65 as a cutoff value, logistic regression analyses were performed for CERAD, AFT, and DSST scores as outcome events, adjusting for covariates including sex, age, race, education, marital status, PIR (poverty income ratio), hypertension, drinking, depression, cardiovascular diseases, stroke, sleep disorders, and smoking. The results showed that after scores exceeded 65, the OR for cognitive impairment for all three scores decreased (Table 3).

**Table 3:** Effect of LE8Score level on CERAD, AFT and DSST: adjusted odds ratios from Segmented Logistic Regression Analysis

	Characteristic	OR^1^	95% CI^1^	*p*-value
CERAD	LE8 Score (< 65)	1.00	(0.98, 1.02)	0.84
	LE8 Score (≥ 65)	0.85	(0.74, 0.99)	0.035
AFT	LE8 Score (< 65)	1.00	(0.98, 1.02)	0.92
	LE8 Score (≥ 65)	0.89	(0.78, 0.99)	0.045
DSST	LE8 Score (< 65)	0.98	(0.96, 1.00)	0.030
	LE8 Score (≥ 65)	0.66	(0.56, 0.78)	< 0.001

^1^OR = Odds Ratio, CI = Confidence Interval

ORs were adjusted for Sex, Age, Race, Education, Marital status, PIR, Hypertension, Drinking, Depression, Cardiovascular diseases, Stroke, Sleep disorder, drinking, and Smoking

## Discussion

In this extensive cross-sectional study utilizing data from the NHANES, the cohort comprised American individuals aged 60 and above. The study employed three cognitive performance metrics: CEARD, AFT, and DSST, to investigate the statistically significant association between LE8 and cognitive impairment. This study demonstrates a significant reduction in cognitive dysfunction, which correlates with elevated levels of LE8. Stratified analysis indicates that the inverse dose-response relationship between LE8 score and cognitive impairment remains consistent across diverse stratification factors.

Our findings on the interrelation between CVH and cognitive impairment partially align with certain aspects of prior studies. Vascular Cognitive Impairment (VCI) denotes the impact of vascular brain lesions on cognitive abilities, ranging from mild to severe impairment [21]. In elderly patients, the decline of bodily functions exacerbates atherosclerosis, which often accumulates in blood vessels across various organs, including the heart, brain, and kidneys. Age-related arterial dysfunction emerges as a critical therapeutic target in managing cardiovascular and cerebrovascular events. Among various manifestations of arterial dysfunction, the stiffness of elastic arteries, such as the aorta and carotid arteries, in the elderly is especially indicative of an increased risk of cardiovascular and cerebrovascular events, as well as Alzheimer’s disease (AD) and other dementias in later life [22–24]. A recent proteomic analysis of patients diagnosed with AD or mild cognitive impairment, who also exhibited intracranial atherosclerosis at autopsy, revealed a potential link to cognitive decline. This association is attributed to synaptic dysregulation, diminished neuroplasticity, and abnormal myelination processes within signaling mechanisms [25].

Hypertension, a critical factor in CVH, significantly influences the pathophysiology of patients suffering from cognitive impairment. This condition facilitates the formation and accumulation of atherosclerotic plaques in the carotid, vertebral, and intracranial cerebral arteries [26]. Cerebral blood vessels possess an intrinsic self-protection mechanism that ensures adequate blood flow by stabilizing arterial circulation [27–29]. Persistent hypertension and atherosclerosis disrupt the regulatory mechanisms of blood and oxygen supply, leading to diminished cerebral perfusion. This metabolic disorder is potentially linked to brain dysfunction and consequent cognitive decline [30, 31]. Additionally, research indicates that hypertension-induced alterations in cognitive function may be associated with the disruption of the blood-brain barrier and imbalances in angiotensin II, interleukin-6 (IL-6), and interleukin-17 (IL-17) [32–34].

Furthermore, the prevalence of cognitive impairment and dementia in elderly patients’ post-stroke, attributable to hypertension and various other causes, may reach up to 15-70% [35–37]. The etiology of post-stroke cognitive impairment (PSCI) is multifaceted, encompassing factors such as large artery disease, cerebral microangiopathy, and pulmonary vascular pathology. A stroke precipitates neurovascular unit dysfunction, a fundamental element of brain parenchyma responsible for brain maintenance. This dysfunction underscores the intricate and interdependent relationship between cerebral vasculature and neural structures [38]. Stroke induces a cascade of pathological events including energy failure, calcium overload, oxidative stress, and blood-brain barrier dysfunction. This sequence culminates in detrimental inflammation at the cerebral microvasculature, resulting in either immediate or progressive neurological dysfunction [39–41].

Prior research has established a significant correlation between cardiovascular diseases (CVD), including atrial fibrillation, coronary heart disease, heart failure, and myocardial infarction, and alterations in cognitive impairment [42–44]. Alterations in cognitive function attributable to CVD could be linked to inflammatory processes. Research indicates that cardiomyocyte demise and the elevation of pro-inflammatory cytokines in the bloodstream, both consequences of CVD, contribute to augmented oxidative stress and systemic inflammation. These effects extend to the brain, precipitating neuronal death and subsequent cognitive decline [45, 46]. Patients exhibiting cognitive dysfunction typically present with diminished dendritic density in the brain. CVD may impair myocardial contractility, leading to decreased cerebral blood flow. Subsequently, cerebral hypoperfusion can induce an overproduction of reactive oxygen species, impairing brain mitochondrial function and promoting brain cell death. This cascade of events is particularly detrimental to synaptic function, culminating in a decline in cognitive abilities [43, 47]. In summary, the pathogenesis of cognitive impairment is multifaceted and intricate. Fundamentally, it is closely associated with the patient’s cardiovascular health. Monitoring cardiovascular health using the LE8 score is particularly crucial in the forthcoming years, as it can significantly aid in the prevention and reduction of cognitive impairment.

Secondly, lifestyle plays an important role in cognitive health. Lifestyle factors included in the LE8 score, such as physical activity, dietary habits, smoking status, and sleep quality, are significantly linked to cognitive function. Physical activity promotes cerebral blood circulation and increases the release of neurotrophic factors, thereby improving cognitive function [48, 49]. A healthy diet, such as the Mediterranean diet, which is rich in antioxidants and anti-inflammatory components, helps reduce brain inflammation and protect neurons [50]. In contrast, smoking and prolonged unhealthy dietary habits increase oxidative stress and inflammation, leading to gradual cognitive decline. Moreover, sleep deprivation has been shown to be related to key pathological features of Alzheimer’s disease, such as amyloid deposition, indicating that sleep is a key factor in maintaining cognitive health [51, 52].

In analyzing the role of education level, income, gender, and LE8 score on cognitive function, a complex interaction among these factors was observed. Education level is considered an important determinant of cognitive reserve, with higher education levels typically enhancing the brain’s resilience and thus slowing the rate of cognitive decline [53, 54]. Income level may have an indirect effect on cognitive health by influencing lifestyle and access to healthcare resources. For example, high-income groups are more likely to have access to healthy foods, physical activity facilities, and quality healthcare, which contribute to higher LE8 scores and, consequently, promote cognitive health. Gender also plays an important moderating role in the relationship between cognitive function and LE8 scores. Research indicates that women exhibit some unique patterns in the relationship between cardiovascular health and cognitive function; for instance, women may be more susceptible to cardiovascular health conditions in middle and old age due to hormonal changes, which also affects their cognitive function. Additionally, women may have different behavior patterns in physical activity and diet compared to men, which may lead to varying impacts on LE8 scores and cognitive function [55–57]. These interactions suggest that considering the LE8 score alone may be insufficient to fully reveal its complexity in relation to cognitive function. Sociodemographic variables such as education, income, and gender not only directly affect cognitive health but also indirectly influence cognitive function by moderating various aspects of the LE8 score. Therefore, when designing prevention and intervention strategies for cognitive impairment, it is important to take these interacting factors into account in order to create more individualized interventions. For instance, improving education levels and increasing access to health resources may be of great significance in enhancing LE8 scores and improving cognitive health.

This article acknowledges several limitations. Primarily, the data on diet, physical activity, nicotine exposure, and sleep health in LE8 were derived from patient self-reports, potentially introducing recall bias. Additionally, despite multivariable adjustments, residual confounding factors may still influence the outcomes. Furthermore, while the study utilized three cognitive evaluation metrics, significant statistical differences were observed in the cognitive scores when LE8 was treated as a continuous variable, yet variations persisted when categorized. Lastly, the study’s sample was exclusively American, raising questions about its broader applicability across diverse racial groups, which warrants further investigation.

## Conclusion

In this nationally representative sample of U.S. adults, LE8 score was strongly inversely associated with cognitive impairment in older patients. LE8 may be effective to prevent cognitive impairment. This result emphasizes that early healthy life intervention for elderly patients to maintain cardiovascular health may be of great significance in preventing cognitive impairment. Future research should focus on exploring the precise mechanism between LE8 and cognitive impairment.

## Electronic supplementary material

Below is the link to the electronic supplementary material.

Supplementary Material 1

## Data Availability

All data can be found at https://wwwn.cdc.gov/nchs/nhanes/Default.aspx.
