# Supplementary material for: Association between life’s essential 8 and cognitive impairment in older patients: results from NHANES 2011–2014
Source: BMC Geriatr. 2024 Nov 14;24:943. doi: 10.1186/s12877-024-05547-4 (PMC11566281; doi:10.1186/s12877-024-05547-4)
Supplement: Supplementary file 1 — Supplementary Material 1 [file 12877_2024_5547_MOESM1_ESM.docx]

Supplementary Table 1. Definition and scoring approach for the American Heart Association’s Life’s Essential 8 score.

| Domain | CVH Metric | Measurement | Quantification and Scoring of CVH Metric |
| --- | --- | --- | --- |
| Health Behaviors | Diet | Healthy Eating Index-2015 diet score percentile | Quantiles of DASH-style diet adherence  **Scoring (Population):**  Points Quantile  100 ≥95^th^ percentile (top/ideal diet)  80 75^th^ – 94^th^ percentile  50 50^th^ – 74^th^ percentile  25 25^th^ – 49^th^ percentile  0 1^st^ – 24^th^ percentile (bottom/least ideal quartile) |
|  | Physical activity | Self-reported minutes of moderate or vigorous physical activity per week | **Metric:** Minutes of moderate (or greater) intensity activity per week  **Scoring:**  Points Minutes  100 ≥150  90 120 – 149  80 90 – 119  60 60 – 89  40 30 – 59  20 1 – 29  0 0 |
|  | Nicotine exposure | Self-reported use of cigarettes or inhaled nicotine- delivery system | **Metric:** Combustible tobacco use and/or inhaled NDS use; or secondhand smoke exposure  **Scoring:**  Points Status  100 Never smoker  75 Former smoker, quit ≥5 yrs  50 Former smoker, quit 1 - <5 yrs  25 Former smoker, quit <1 year, or currently using inhaled NDS  0 Current smoker  Subtract 20 points (unless score is 0) for living with active indoor smoker in home |
|  | Sleep health | Self-reported average hours of sleep per night | **Metric:** Average hours of sleep per night  **Scoring:**  Points Level  100 7 – <9  90 9 – <10  70 6 – <7  40 5 – <6 or ≥10  20 4 – <5  0 <4 |
| Health Factors | Body mass index | Body weight (kg) divided by height squared (m^2^) | **Metric:** Body mass index (kg/m^2^)  **Scoring:** Points Level 100 <25  70 25.0 – 29.9  30 30.0 – 34.9  15 35.0 – 39.9  0 ≥40.0 |
|  | Blood lipids | Plasma total and HDL-cholesterol with calculation of non-HDL-cholesterol | **Metric:** Non-HDL-cholesterol (mg/dL)  **Scoring:**  Points Level  100 <130  60 130 – 159  40 160 – 189  20 190 – 219  0 ≥220  If drug-treated level, subtract 20 points |
|  | Blood glucose | Fasting blood glucose or casual hemoglobin A1c | **Metric:** Fasting blood glucose (mg/dL) or Hemoglobin A1c (%)  **Scoring:**  Points Level  100 No history of diabetes and FBG <100 (or HbA1c < 5.7)  60 No diabetes and FBG 100 – 125 (or HbA1c 5.7-6.4) (Pre-diabetes)  40 Diabetes with HbA1c <7.0  30 Diabetes with HbA1c 7.0 – 7.9  20 Diabetes with HbA1c 8.0 – 8.9  10 Diabetes with Hb A1c 9.0 – 9.9  0 Diabetes with HbA1c ≥10.0 |
|  | Blood pressure | Appropriately measured systolic and diastolic blood pressure | **Metric:** Systolic and diastolic blood pressure (mm Hg)  **Scoring:**  Points Level  100 <120/<80 (Optimal)  75 120-129/<80 (Elevated)  50 130-139 or 80-89 (Stage I HTN)  25 140-159 or 90-99  0 ≥160 or ≥100  Subtract 20 points if treated level |

**Reference**

1. Lloyd-Jones DM, Allen NB, Anderson CAM, et al. Life's Essential 8: Updating and Enhancing the American Heart Association's Construct of Cardiovascular Health: A Presidential Advisory From the American Heart Association. *Circulation*. Aug 2 2022;146(5):e18-e43.
2. Lloyd-Jones DM, Ning H, Labarthe D, et al. Status of Cardiovascular Health in US Adults and Children Using the American Heart Association's New "Life's Essential 8" Metrics: Prevalence Estimates From the National Health and Nutrition Examination Survey (NHANES), 2013 Through 2018. *Circulation*. Sep 13 2022;146(11):822-835.

Supplementary Table 2. Healthy Eating Index-2015 Components & Scoring Standards^1^

| Component | Maximum points | Standard for maximum score | Standard for minimum score of zero |
| --- | --- | --- | --- |
| *Adequacy* | | | |
| Total Fruits[^2^](https://epi.grants.cancer.gov/hei/developing.html#f2) | 5 | ≥0.8 cup equiv. per 1,000 kcal | No Fruit |
| Whole Fruits[^3^](https://epi.grants.cancer.gov/hei/developing.html#f3) | 5 | ≥0.4 cup equiv. per 1,000 kcal | No Whole Fruit |
| Total Vegetables[^4^](https://epi.grants.cancer.gov/hei/developing.html#f4) | 5 | ≥1.1 cup equiv. per 1,000 kcal | No Vegetables |
| Greens and Beans[^4^](https://epi.grants.cancer.gov/hei/developing.html#f4) | 5 | ≥0.2 cup equiv. per 1,000 kcal | No Dark Green Vegetables or Legumes |
| Whole Grains | 10 | ≥1.5 oz equiv. per 1,000 kcal | No Whole Grains |
| Dairy[^5^](https://epi.grants.cancer.gov/hei/developing.html#f5) | 10 | ≥1.3 cup equiv. per 1,000 kcal | No Dairy |
| Total Protein Foods[^6^](https://epi.grants.cancer.gov/hei/developing.html#f6) | 5 | ≥2.5 oz equiv. per 1,000 kcal | No Protein Foods |
| Seafood and Plant Proteins[^6^](https://epi.grants.cancer.gov/hei/developing.html#f6)^,^[^7^](https://epi.grants.cancer.gov/hei/developing.html#f7) | 5 | ≥0.8 oz equiv. per 1,000 kcal | No Seafood or Plant Proteins |
| Fatty Acids[^8^](https://epi.grants.cancer.gov/hei/developing.html#f8) | 10 | (PUFAs + MUFAs)/SFAs ≥2.5 | (PUFAs + MUFAs)/SFAs ≤1.2 |
| *Moderation* | | | |
| Refined Grains | 10 | ≤1.8 oz equiv. per 1,000 kcal | ≥4.3 oz equiv. per 1,000 kcal |
| Sodium | 10 | ≤1.1 gram per 1,000 kcal | ≥2.0 grams per 1,000 kcal |
| Added Sugars | 10 | ≤6.5% of energy | ≥26% of energy |
| Saturated Fats | 10 | ≤8% of energy | ≥16% of energy |

**(1)** Intakes between the minimum and maximum standards are scored proportionately.

**(2)** Includes 100% fruit juice.

**(3)** Includes all forms except juice.

**(4)** Includes legumes (beans and peas).

**(5)** Includes all milk products, such as fluid milk, yogurt, and cheese, and fortified soy beverages.

**(6)** Includes legumes (beans and peas).

**(7)** Includes seafood, nuts, seeds, soy products (other than beverages), and legumes (beans and peas).

**(8)** Ratio of poly- and monounsaturated fatty acids (PUFAs and MUFAs) to saturated fatty acids (SFAs).

y elements that are encouraged. For these components, higher scores reflect higher intakes, because higher intakes are desirable.

*Moderation components* represent the food groups and dietary elements for which there are recommended limits to consumption. For moderation components, higher scores reflect lower intakes, because lower intakes are more desirable.

**Reference**

1. Krebs-Smith SM, Pannucci TE, Subar AF, et al. Update of the Healthy Eating Index: HEI-2015. J Acad Nutr Diet. Sep 2018;118(9):1591-1602.

2. National Cancer Institute. HEI Scoring Algorithm. Accessed August, 2022. <https://epi.grants.cancer.gov/hei/hei-scoring-method.html>

Supplementary Table 3. Subgroup analysis of the relationship between LE8 score and CERAD

|  | Cardiovascular health group | | |  |  |
| --- | --- | --- | --- | --- | --- |
|  | Low | Moderate | High | P for trend | P for interaction |
| Age |  |  |  |  | 0.001 |
| 60-75 | Reference | 0.909(0.650-1.272) | 0.359(0.206-0.627) | 0.001 |  |
| ＞75 | Reference | 1.715(0.841-3.500) | 1.259(0.517-3.063) | 0.230 |  |
| Sex, n (%) |  |  |  |  | 0.012 |
| Male | Reference | 0.991(0.658-1.493) | 0.578(0.324-1.031) | 0.065 |  |
| Female | Reference | 1.067(0.677-1.682) | 0.378(0.175-0.818) | 0.009 |  |
| Race, n (%) |  |  |  |  | 0.053 |
| Mexican American | Reference | 1.005(0.425-2.380) | 0.487(0.110-2.151) | 0.548 |  |
| Other Hispanic | Reference | 0.836(0.374-1.867) | 0.378(0.087-1.630) | 0.417 |  |
| Non-Hispanic White | Reference | 1.225(0.722-2.080) | 0.690(0.342-1.393) | 0.082 |  |
| Non-Hispanic Black | Reference | 1.061(0.632-1.783) | 0.592(0.221-1.601) | 0.450 |  |
| Other Race - Including Multi- Racial | Reference | 2.123(0.253-17.817) | 0.972(0.098-9.645) | 0.262 |  |
| Marital status, n (%) |  |  |  |  | 0.522 |
| Married/Living with Partner | Reference | 1.061(0.692-1.629) | 0.530(0.294-0.956) | 0.011 |  |
| Never married | Reference | 1.580(0.491-5.089) | 2.361(0.505-11.049) | 0.550 |  |
| Widowed/Divorced/ Separated | Reference | 0.968(0.611-1.535) | 0.301(0.118-0.770) | 0.026 |  |
| Education levels, n (%) |  |  |  |  | 0.001 |
| < High school | Reference | 1.089(0.680-1.746) | 0.716(0.294-1.741) | 0.567 |  |
| High school | Reference | 1.404(0.757-2.603) | 1.016(0.373-2.764) | 0.453 |  |
| College or above | Reference | 1.069(0.611-1.869) | 0.613(0.302-1.248) | 0.098 |  |
| Income status |  |  |  |  | 0.026 |
| High income | Reference | 0.650(0.300-1.314) | 0.460(0.194-1.090) | 0.210 |  |
| Middle income | Reference | 1.281(0.735-2.231) | 0.518(0.210-1.274) | 0.049 |  |
| Low income | Reference | 1.304(0.453-3.753) | 0.713(0.147-3.453) | 0.607 |  |
| Smoking, n (%) |  |  |  |  | 0.757 |
| Yes | Reference | 1.020(0.706-1.474) | 0.524(0.256-1.073) | 0.134 |  |
| No | Reference | 1.065(0.617-1.839) | 0.507(0.258-0.999) | 0.006 |  |
| Drinking, n (%) |  |  |  |  | 0.093 |
| Yes | Reference | 0.965(0.673-1.385) | 0.619(0.370-1.036) | 0.096 |  |
| No | Reference | 1.235(0.710-2.148) | 0.255(0.090-0.725) | 0.003 |  |
| Sleep Disorder, n (%) |  |  |  |  | 0.763 |
| Yes | Reference | 0.979(0.622-1.541) | 0.355(0.101-1.241) | 0.249 |  |
| No | Reference | 1.035(0.683-1.568) | 0.512(0.297-0.882) | 0.002 |  |
| Stroke, n (%) |  |  |  |  | 0.076 |
| Yes | Reference | 1.520(0.559-4.133) | 0.283(0.030-2.706) | 0.222 |  |
| No | Reference | 1.014(0.738-1.393) | 0.532(0.333-0.848) | 0.004 |  |
| Cardiovascular diseases, n (%) |  |  |  |  | 0.244 |
| Yes | Reference | 0.821(0.446-1.511) | 0.471(0.157-1.409) | 0.403 |  |
| No | Reference | 1.126(0.793-1.597) | 0.540(0.326-0.896) | 0.002 |  |
| Hypertension, n (%) |  |  |  |  | 0.368 |
| Yes | Reference | 1.049(0.738-1.492) | 0.460(0.232-0.911) | 0.032 |  |
| No | Reference | 1.043(0.573-1.901) | 0.557(0.270-1.146) | 0.034 |  |

Supplementary Table 4. Subgroup analysis of the relationship between LE8 score and AFT

|  | Cardiovascular health group | | |  |  |
| --- | --- | --- | --- | --- | --- |
|  | Low | Moderate | High | P for trend | P for interaction |
| Age |  |  |  |  | 0.139 |
| 60-75 | Reference | 0.767(0.544-1.081) | 0.624(0.380-1.024) | 0.153 |  |
| ＞75 | Reference | 0.748(0.398-1.406) | 0.750(0.330-1.703) | 0.662 |  |
| Sex, n (%) |  |  |  |  | 0.428 |
| Male | Reference | 0.677(0.439-1.042) | 0.711(0.396-1.275) | 0.208 |  |
| Female | Reference | 0.867(0.570-1.319) | 0.611(0.331-1.129) | 0.277 |  |
| Race, n (%) |  |  |  |  | 0.101 |
| Mexican American | Reference | 1.014(0.355-2.899) | 1.533(0.346-6.794) | 0.792 |  |
| Other Hispanic | Reference | 0.329(0.149-0.727) | 0.062(0.007-0.535) | 0.005 |  |
| Non-Hispanic White | Reference | 0.926(0.511-1.678) | 0.810(0.378-1.732) | 0.850 |  |
| Non-Hispanic Black | Reference | 0.935(0.581-1.505) | 0.968(0.432-2.168) | 0.961 |  |
| Other Race - Including Multi- Racial | Reference | 0.938(0.496-1.648) | 0.681(0.345-1.297) | 0.749 |  |
| Marital status, n (%) |  |  |  |  | 0.899 |
| Married/Living with Partner | Reference | 0.786(0.507-1.219) | 0.776(0.444-1.357) | 0.549 |  |
| Never married | Reference | 1.465(0.395-5.441) | 1.000(0.145-6.907) | 0.779 |  |
| Widowed/Divorced/ Separated | Reference | 0.717(0.461-1.117) | 0.533(0.254-1.117) | 0.189 |  |
| Education levels, n (%) |  |  |  |  | 0.001 |
| < High school | Reference | 0.608(0.382-0.969) | 0.479(0.193-1.190) | 0.083 |  |
| High school | Reference | 0.896(0.511-1.572) | 1.241(0.52-2.954) | 0.658 |  |
| College or above | Reference | 1.499(0.737-3.049) | 1.741(0.788-3.849) | 0.391 |  |
| Income status |  |  |  |  | 0.001 |
| High income | Reference | 0.846(0.366-1.954) | 0.727(0.273-1.936) | 0.806 |  |
| Middle income | Reference | 0.867(0.506-1.487) | 0.659(0.427-1.829) | 0..656 |  |
| Low income | Reference | 0.738(0.471-1.157) | 0.703(0.316-1.564) | 0.391 |  |
| Smoking, n (%) |  |  |  |  | 0.174 |
| Yes | Reference | 0.638(0.444-0.916) | 0.588(0.305-1.135) | 0.043 |  |
| No | Reference | 1.107(0.617-1.985) | 0.895(0.454-1.763) | 0.584 |  |
| Drinking, n (%) |  |  |  |  | 0.057 |
| Yes | Reference | 0.724(0.499-1.051) | 0.657(0.391-1.104) | 0.185 |  |
| No | Reference | 0.837(0.500-1.402) | 0.696(0.336-1.442) | 0.618 |  |
| Sleep Disorder, n (%) |  |  |  |  | 0.686 |
| Yes | Reference | 0.873(0.553-1.378) | 0.951(0.377-2.395) | 0.836 |  |
| No | Reference | 0.684(0.455-1.028) | 0.574(0.343-0.961) | 0.096 |  |
| Stroke, n (%) |  |  |  |  | 0.201 |
| Yes | Reference | 0.622(0.247-1.565) | 0.346(0.060-1.981) | 0.424 |  |
| No | Reference | 0.796(0.578-1.097) | 0.720(0.464-1.118) | 0.284 |  |
| Cardiovascular diseases, n (%) |  |  |  |  | 0.804 |
| Yes | Reference | 0.871(0.462-1.644) | 0.833(0.462-1.644) | 0.902 |  |
| No | Reference | 0.746(0.530-1.050) | 0.641(0.402-1.024) | 0.142 |  |
| Hypertension, n (%) |  |  |  |  | 0.108 |
| Yes | Reference | 0.830(0.587-1.172) | 0.709(0.393-1.279) | 0.442 |  |
| No | Reference | 0.712(0.382-1.327) | 0.733(0.358-1.503) | 0.565 |  |

Supplementary Table 5. Subgroup analysis of the relationship between LE8 score and DSST

|  | Cardiovascular health group | | |  |  |
| --- | --- | --- | --- | --- | --- |
|  | Low | Moderate | High | P for trend | P for interaction |
| Age |  |  |  |  | 0.569 |
| 60-75 | Reference | 0.690(0.506-0.942) | 0.181(0.100-0.330) | 0.001 |  |
| ＞75 | Reference | 0.532(0.298-0.950) | 0.102(0.003-0.321) | 0.001 |  |
| Sex, n (%) |  |  |  |  | 0.053 |
| Male | Reference | 0.795(0.536-1.180) | 0.218(0.110-0.430) | 0.001 |  |
| Female | Reference | 0.520(0.354-0.762) | 0.101(0.041-0.245) | 0.001 |  |
| Race, n (%) |  |  |  |  | 0.540 |
| Mexican American | Reference | 0.643(0.286-1.442) | 0.394(0.108-1.434) | 0.342 |  |
| Other Hispanic | Reference | 0.907(0.417-1.973) | 0.560(0.161-1.949) | 0.631 |  |
| Non-Hispanic White | Reference | 0.641(0.372-1.104) | 0.104(0.030-0.362) | 0.002 |  |
| Non-Hispanic Black | Reference | 0.792(0.504-1.242) | 0.158(0.030-0.362) | 0.005 |  |
| Other Race - Including Multi- Racial | Reference | 0.839(0.628-1.357) | 0.367(0.069-0.105) | 0.221 |  |
| Marital status, n (%) |  |  |  |  | 0.421 |
| Married/Living with Partner | Reference | 0.682(0.457-1.019) | 0.180(0.090-0.361) | 0.001 |  |
| Never married | Reference | 0.663(0.250-1.757) | 0.271(0.048-1.538) | 0.001 |  |
| Widowed/Divorced/ Separated | Reference | 0.673(0.446-1.016) | 0.134(0.050-0.359) | 0.001 |  |
| Education levels, n (%) |  |  |  |  | 0.001 |
| < High school | Reference | 0.630(0.393-1.011) | 0.510(0.223-1.168) | 0.120 |  |
| High school | Reference | 1.062(0.594-1.897) | 0.197(0.043-0.902) | 0.072 |  |
| College or above | Reference | 0.620(0.344-1.117) | 0.064(0.014-0.284) | 0.001 |  |
| Income status |  |  |  |  | 0.001 |
| High income | Reference | 0.690(0.280-1.702) | 0.061(0.007-0.516) | 0.037 |  |
| Middle income | Reference | 0.507(0.319-0.807) | 0.133(0.049-1.048) | 0.001 |  |
| Low income | Reference | 1.132(0.738-1.738) | 0.465(0.206-1.048) | 0.057 |  |
| Smoking, n (%) |  |  |  |  | 0.098 |
| Yes | Reference | 0.677(0.485-0.946) | 0.193(0.084-0.443) | 0.001 |  |
| No | Reference | 0.623(0.381-1.019) | 0.142(0.067-0.301) | 0.001 |  |
| Drinking, n (%) |  |  |  |  |  |
| Yes | Reference | 0.702(0.500-0.986) | 0.195(0.104-0.365) | 0.001 |  |
| No | Reference | 0.555(0.346-0.888) | 0.103(0.038-0.281) | 0.001 |  |
| Sleep Disorder, n (%) |  |  |  |  | 0.266 |
| Yes | Reference | 0.575(0.378-0.875) | 0.311(0.103-0.938) | 0.013 |  |
| No | Reference | 0.645(0.444-0.937) | 0.129(0.069-0.243) | 0.001 |  |
| Stroke, n (%) |  |  |  |  | 0.291 |
| Yes | Reference | 0.827(0.337-2.032) | 0.289(0.051-1.646) | 0.367 |  |
| No | Reference | 0.644(0.482-0.859) | 0.155(0.089-0.271) | 0.001 |  |
| Cardiovascular diseases, n (%) |  |  |  |  | 0.431 |
| Yes | Reference | 0.545(0.315-0.944) | 0.085(0.019-0.386) | 0.003 |  |
| No | Reference | 0710(0.516-0.977) | 0.188(0.106-0.334) | 0.001 |  |
| Hypertension, n (%) |  |  |  |  | 0.105 |
| Yes | Reference | 0.771(0.558-1.065) | 0.145(0.060-0.348) | 0.001 |  |
| No | Reference | 0.439(0.260-0.739) | 0.135(0.064-0.284) | 0.001 |  |
